# Ozone and Bioactive Compounds in Grapes and Wine

**DOI:** 10.3390/foods10122934

**Published:** 2021-11-28

**Authors:** Margherita Modesti, Monica Macaluso, Isabella Taglieri, Andrea Bellincontro, Chiara Sanmartin

**Affiliations:** 1 Department for Innovation in Biological, Agro-Food and Forest Systems, University of Tuscia, Via S. Camillo de Lellis, 01100 Viterbo, Italy; bellin@unitus.it; 2 Department of Agriculture, Food and Environment, University of Pisa, Via del Borghetto 80, 56124 Pisa, Italy; monica.macaluso@phd.unipi.it (M.M.); chiara.sanmartin@unipi.it (C.S.); 3 Interdepartmental Research Center “Nutraceuticals and Food for Health”, University of Pisa, Via del Borghetto 80, 56124 Pisa, Italy

**Keywords:** ozonisation, antioxidants, elicitation, table grape, wine grape, wine

## Abstract

Ozone is widely used in the agri-food and food processing industries mainly as a sanitizing agent. However, it has recently become clear that ozone exposition leads to another important benefit: in living tissues, the induced-oxidative stress triggers the antioxidant response, and, therefore, it enhances the production of antioxidant and stress-related secondary metabolites. As such, ozone can be considered an abiotic elicitor. The goal of the present review was to critically summarize knowledge about the possibility of improving bioactive compounds and, consequently, the health-related properties of grapes and wine, by using ozone. The greatest interest has been given not only to the pre- and post-harvest treatment of table and wine grapes, but also to the explanation of the mechanisms involved in the ozone-related response and the main secondary metabolites biosynthetic pathways. From the literature available, it is clear that the effect of ozone treatment on health-related properties and secondary metabolites accumulation depends on many factors, such as the cultivar, but also the form (water or gaseous), doses, and application method of ozone. Most of the published papers report an increase in antioxidant compounds (e.g., polyphenols) and stress-related volatiles, confirming the hypothesis that ozone could be used to improve berry and wine compositional and sensory quality.

## 1. Introduction

Ozone (O_3_) is the gaseous triatomic molecule of oxygen, naturally part of the stratosphere [1,2]. O_3_ has a strong oxidative potential, which makes it highly instable [2,3], and leads to a rapid oxidation of any organic matter (such as fungi, bacteria, yeasts, and viruses) which it is in contact with, and a rapid reconversion to O_2_ without the production of any harmful by-products [3]. In 2001, O_3_ was identified and generally recognized as a safe (GRAS) substance by the U.S. Food and Drug Administration (FDA), and it has, therefore, been widely used in the food industry [4]. Its use in the agri-food sector represents an environmental and human health friendly approach to sanitize and to preserve fresh food. Its application on fresh products has revealed many advantages, such as the reduction of post-harvest disease development, reduction of spore production, oxidation of ethylene, and the slowing down of fruit respiration and, in general, the ripening process, thus, overall increasing the shelf-life [1,5,6].

Many patents have been developed for O_3_ application on plants, fruits, and vegetables. The first method was patented in 1988 by Cantelli [7], who developed a method based on the storage of fresh products in closed containers with a constant O_3_ concentration of 0.05 ppm. In 1990, Karg [8] set up the sterilization of minimally processed fruits and vegetables from different contaminants using O_3_ mixed with other gases (CO_2_ and N_2_) within the processing and packaging rooms [9]. Finally, in 2012, the first application in the wine industry was proposed by Mencarelli and Catelli [10]. The patented method, called Purovino^®^, involves the use of O_3_ with the goal to decontaminate grapes and winery facilities during winemaking, by reducing the employ of sulphur dioxide. 

In addition, O_3_ exposure of plants and harvested fruits and vegetables has been demonstrated to induce important metabolic shifts. Hence, it can promote the biosynthesis of secondary metabolites, such as polyphenols and volatile organic compounds (VOCs), in plants and in plants products. The resulted metabolic shifts are strictly related to different factors, such as concentration and length of exposure, and are cultivar-dependent [11,12,13,14].

Therefore, in the recent years, many studies have focused on the possibility to use O_3_ as an abiotic stressor to trigger the content of bioactive compounds in plants and plant products [2,15,16,17,18]. The following paragraphs will focus on the main secondary metabolic responses induced in vines and grapes after O_3_ exposition, focusing on those metabolites important for grapes and wine quality, as well as on their health-promoting properties. The most relevant papers selected and discussed in the present review are summarized in Table 1. 

## 2. Review Methodology

The bibliographic identification was conducted between December 2020 and August 2021 using relevant electronic bibliographic databases (e.g., Web of Science, Science Direct, and PubMed) to ensure the highest coverage for significant papers. The primary keywords (ozone, table and wine grapes, wine) were combined using the set operator AND with secondary keywords (i.e., pre- and post-harvest treatment, bioactive compounds, volatile organic compounds, polyphenols, and antioxidants). To find and select documents, recently published reviews were firstly analysed. Then, starting from pre-selected papers, literature older than the mentioned time period was included if considered helpful to improve topic description. Authors independently evaluated the available literature using predefined eligibility criteria, resolving disagreement by discussion. To restrict and focus the aim of the research, only papers sections dealing with polyphenols and volatiles following ozone treatments were selected (excluding sections dealing with other grapes and wine traits). A total of 88 papers were selected in the end. 

## 3. Ozone and Plant Interaction

### 3.1. Reactive Oxygen Species Production in Plant Tissue 

Once O_3_ penetrates the cells, it is immediately converted in reactive oxygen species (ROS), and, therefore, an endogenous ROS production, known as *oxidative burst*, contributes to the overall oxidizing potential of O_3_ [34]. ROS production has been correlated with cellular stress responses which, consequentially, leads to important shifts in secondary metabolism [35]. The oxidative stress is characterized by an imbalance of the cellular redox status, and shifts of cell metabolism are needed to rebalance the basal levels of ROS [36]. The scavenging antioxidant system is characterized by the activity of specific enzymes having an antioxidant role, and by the antioxidant compound biosynthesis (stress-related volatiles, such as C6 (aldehydes, alcohols, and esters) and terpenoids and polyphenols, stilbenes, isoprene, and ascorbic acid) [37,38].

Secondary metabolites are biologically active compounds produced under specific condition in plants tissues, and are generally involved in plants adaptation as a response to changes in external condition. Several secondary metabolites (such as polyphenols, flavonols and tannins, essential oils, sterols, phytoalexins, and monoterpenes) have been demonstrated to have important functional effects on human health, and, therefore, techniques aimed at increasing their content in plants and plant products are becoming popular [39,40]. Elicitation can be defined as a controlled stress induced by elicitors which leads to the production of secondary metabolites [41,42], and can, therefore, improve the biological activity of plant products. Given the well-established efficacy of O_3_ for the agri-food preservation and in inducing secondary metabolism shifts, recently, O_3_ has been suggested as a pre-and post-harvest elicitor [16,19,43,44]. 

### 3.2. Ozone and Polyphenols

Grape and wine polyphenols have considerable importance not only for their contribution to wine quality parameters, such as color, flavor, astringency, bitterness, and ageing behavior [45], but also for their antioxidant capacity. In particular, phenolic acids, flavonoids, anthocyanins, and tannins are well-known to have many health benefits [5,20,46] due to their activity as radical scavengers. 

Given the antioxidant role that polyphenols play in the cells, most of them are biosynthesized in both vines and grapes as a response to biotic and abiotic stresses thanks to the activation of the phenylpropanoid pathway [47]. Researchers have indeed observed that a higher polyphenol accumulation is due to the increase of activity of enzymes, such as phenylalanine ammonia lyase (PAL), stilbene synthases (STS), and flavonol synthases (FLS) [5,48,49]. The key point is that O_3_, playing as stressor, induces the defence mechanism to protect plants and fruits tissue against oxidative stress-related damages. 

However, the effect on polyphenol content after ozonisation is not always clear [5,40]: some papers describe a positive effect of O_3_ in terms of polyphenols accumulation [11,15,29], whereas others talk about negative ones, especially their oxidation with consequent reduction [18,50,51]. 

The effect of O_3_ in inducing the biosynthesis of polyphenols mainly depends on the concentration and method, as well as the length of exposure. Generally, high ozone doses lead to an over oxidation which will induce a phenol decrease [18,51]. Nevertheless, a more controlled and limited amount of O_3_ results in a controlled oxidative stress which may stimulate the biosynthesis of these compounds [52].

Apart from the doses utilized, the method of application is another crucial aspect which can influence the effects and the consequences of the ozonisation process. O_3_ in gaseous form is much more stable and effective in potentially inducing the oxidation. On the contrary, ozonated water is less stable and less oxidative, and, therefore, a negative impact on phenol accumulation is unlikely to occur [53]. Lastly, the duration and the type of exposition (single, continuous, or intermittent) also matters. For example, it has been observed that an overnight treatment of wine grapes with O_3_ at 1.5 g/L increases phenol and anthocyanin content, whereas longer and continues exposition decreases phenolics [11].

### 3.3. Ozone and Volatile Organic Compounds

Volatiles are important secondary metabolites which are often involved in defence mechanisms. VOCs are indeed produced in, and released from, leaves, flowers, and fruits with the main functions of: (i) attracting pollinators and beneficial insects; (ii) protecting plants against pathogen infection and herbivore attack; (iii) creating molecules signal for plant–plant communication [54]. Because of these roles, VOCs are often synthesized under stressed condition. Specifically, in the case of the oxidative stress, the mechanism is still linked with ROS production, which determines the membrane lipid peroxidation [55] and VOCs associated with lipid peroxidation. Compounds, such as C6 (mainly aldehydes and alcohols), methanol, and methyl salicylate, are monitored as signalling volatiles [56], and have antibacterial and fungicidal properties [56]. 

In living tissue, such as vine leaves and grapes, stress responses occur when O_3_ is applied, especially if accurately managed, and stress-related VOCs biosynthesis generally is induced [57,58]. Remarkably, C6 volatiles (especially aldehydes and alcohols) formed in grapes are generally associated with herbaceous aroma and flavour. Additionally, when C6 volatiles are converted in their acetate esters, the result is pleasant fruity nuances [34]. 

Another class of VOCs known to play a key role in the antioxidant response, and, therefore, also produced after O_3_ exposition, belongs to the terpenoid family, i.e., isoprene, monoterpenes, and sesquiterpenes [59]. The stimulation of terpenoid biosynthesis in fruits and vegetables is extremely significant, considering the crucial role they play in the floral and fruity aroma and taste. Two different pathways are involved in terpenoid biosynthesis: the methylerythritol phosphate (MEP) and the mevalonate (MVE) pathway [60], which are known to be strongly influenced by biotic and abiotic stresses [57,61,62,63]. Several fruits, vegetables, and plant tissues show an increase of terpenoid biosynthesis as a response to ozonisation [14,38,57,64]. It has been hypothesized that terpenes biosynthesis is induced by the oxidative stress because they might act as ROS scavengers, reducing their reactivity [65]. In addition, in O_3_ stressed plants tissues, the inhibition of terpenes biosynthesis results in higher oxidative damage [38], supporting the hypothesis of an antioxidant role, as suggested by Calogirou, 1999 [66]. 

Lastly, another defence mechanism induced by oxidative stress is the stimulation of the activity of different enzymes, including uridine5′-diphospho-gluconosyltransferases (UGTs), which play an indirect role in ROS-detoxification [67]. Glycosylation allows compartmentalization of small and toxic/reactive molecules, such as ROS, but also VOCs, by reducing their volatility through derivatization [68]. As such, the UGTs plays a key role not only in plant defence mechanisms [69], but also in the formation of glycosylated volatiles. Therefore, the induced oxidative stress stimulating UGTs activity can, in turn, increase glycosylated volatiles [70], also known as aromatic precursors. 

## 4. Ozone and Bioactive Compounds in Table and Wine Grape

### 4.1. Table Grape

Table grapes (*Vitis vinifera* L.) are affected in postharvest life by evident quality depletion mainly caused by loss of water, berry softening, browning, and microbiological contamination, the latter mainly due to grey mold action [45]. As stated by many researches, O_3_ treatment ranging from 0.1 mg/L/day or higher allows to prolong table grape shelf-life, inhibiting the growth of grey mold [71,72]. Furthermore, O_3_ was reported to boost the phenolic and aromatic compound biosynthesis, since O_3_ induces in living tissues different defense mechanisms at the genetic, transcriptional, and biochemical level [73].

Researchers have evaluated the effect of O_3_ atmosphere on Napoleón grapes both packed in a macro-perforated polypropylene basket during 38 days of storage at 0 °C under 0.1 mg/L of O_3_, and stored in a 2.5 L glass jar with 8 mg/L of O_3_ flushed for 30 min every 2.5 h. They observed an increase of total stilbenoids for both O_3_ treatments, even if the O_3_ concentrations were not enough to avoid *Botrytis cinerea* spread [19,48,74].

A similar study [20] tested different O_3_ treatments (continuous: 0.1 µL/L; discontinuous. 8 µL/L for 30 min every 2.5 h) on the quality of Autumn Seedless grapes both after protracted storage at low temperature (60 days; 0 °C), and after one week at retail conditions (15 °C). Either continuous or discontinuous treatments, at low temperature, determined an increase of total flavan-3-ol. Furthermore, continuous treatment also retained the initial content of hydroxycinnamates. After the retail period, a significant increase of total polyphenols was observed for both treatments [20].

Cayuela et al., [74] treated two white table grapes (Superior Seedless and Regina Victoria) varieties and one red grape (Cardinal CL80) variety with continuous and discontinuous (12 h/days) O_3_ flows (2 mg/L) during storage (5 °C; 72 days). The shelf-life of O_3_-treated grapes, regardless of the method of application, was significantly prolonged in comparison with the control grapes stored in air [74]. Additionally, discontinuous treatment led to the highest resveratrol content. On the other hand, continuous application induced a reduction of these compounds. In this study, the authors suggested that the continuous presence of O_3_ blocked the resveratrol biosynthesis, whereas a discontinuous action could trigger its biosynthesis [74]. This was also observed by Artés-Hernández et al. and Tomás-Barberán et al. [19,48], where the discontinuous treatment (8 mg/L O_3_ for 30 min, every 2.5 h) boosted the resveratrol content in Napoléon grapes. However, some discrepancies in the results have been evidenced in the literature, probably as a consequence of a cultivar [20,48] or dose-dependent effect of O_3_ treatments: continuous highly concentrated (2 mg/L) treatment could deplete antioxidant compounds as a defensive mechanism toward oxidative stress [75], whereas discontinuous applications could improve their accumulation [21]. 

Admane et al. [22] evaluated the effects of O_3_ pre-treatments at three different concentrations (5, 10, and 20 μL/L on harvested organic Scarlotta table grapes packed under modified atmosphere packaging (MAP) (2% O_2_ and 5% CO_2_), monitoring the quality decay trend, sensory traits, and antioxidant compounds profile during 45 days at 0 ± 0.5 °C, followed by 7 days at higher temperature (15 ± 1.0 °C). The results showed a higher level of polyphenols and antioxidant capacity for all samples O_3_-treated, confirming the O_3_ activity as an elicitor of phenolic compounds biosynthesis. Moreover, the content of anthocyanins in the berry skins of O_3_-treated grapes was significantly higher than in the control ones [22]. The increase of anthocyanins content was also observed in Cardinal grapes stored for 12 days at low temperature [76]. 

Silveira et al. [23] observed similar results in Thompson Seedless and Black Seedless grapes, previously immersed in ozonated water at different concentrations (2, 4, 6, or 8 mg/L) for 4 min at 5 °C, and then stored for 21 days at 5 °C. Namely, a 23–50% and 18.5–28% improvement of total polyphenols was observed in Thompson Seedless and Black Seedless grapes, respectively. Furthermore, all the O_3_ treatments determined a doubling of the antioxidant capacity in Thompson samples, whereas only the treatment with 6 and 8 mg/L increased antioxidant activity in Black Seedless grapes [23].

### 4.2. Wine Grape 

As previously described, O_3_ exposure can cause modifications in grape secondary metabolism, improving the synthesis of phenolics such as stilbenes and anthocyanins [26,77]. 

Paissoni et al. [26] observed that O_3_ treatments for 24 and 72 h affected the initial phases of skin maceration in red vinification for both Barbera and Nebbiolo grapes, favoring the extraction of di-substituted anthocyanins in Nebbiolo grapes. Namely, O_3_ treatment did not affect the final individual anthocyanin extractability, thus, the varietal anthocyanin fingerprint was maintained. O_3_ also influenced the flavanol extraction, which was slowed down in both varieties. In another study, Bellincontro et al. [15] reported a faster fermentation rate, as well as a higher anthocyanin concentration by an overnight O_3_ treatment of Petit Verdot grapes. 

Valetta et al. [27] observed that O_3_ treatment did not activate the stilbene synthesis in Maturano white grapes, even if the leafy chlorogenic acid content was increased; thus, they proposed chlorogenic acid as a biochemical marker of O_3_-induced stress in the *V. vinifera* plant. 

Short-term treatments with O_3_ on fresh grapes have proven effective in determining changes in flavanol fraction, with a significant increase in catechins, and a slightly decreased epicatechin [28]. As suggested by Carbone and Mencarelli [28], the triggering of low-molecular-weight antioxidant biosynthesis could be considered as a defense mechanism against the O_3_-induced oxidative stress. 

At the same time, O_3_ could play a protective role against the oxidation of flavanols since, during post-harvest partial grape dehydration, O_3_ exposure both promotes antioxidant enzymes, and inhibits the oxidant activity of polyphenol oxidase and lipoxygenase enzymes [29]. Botondi et al. [11] showed that O_3_ fumigation at 1.5 g/h for 18 h in continuous flow (shock treatment) could preserve the polyphenol and anthocyanin content. On the other hand, a long-term O_3_ treatment (1.5 g/h, continuous flow, subsequent dehydration with 0.5 g/h of O_3_, 4 h per day) determined a significant oxidation of the polyphenol content. 

Both dehydration and O_3_ effects are cultivar-dependent. As reported by Rìo Segade et al. [77], in Barbera skins, the combination of the two post-harvest stresses (e.g., oxidative and water stress) determined a limited proanthocyanidin loss, together with increased prodelphinidin and limited galloylation percentages. Besides, in Nebbiolo skin, richer in proanthocyanidin, an increased galloylation was observed during dehydration when associated with O_3_ treatment [30]. 

Another study [77] carried out on Moscato Bianco wine grapes (*Vitis vinifera* L.) showed that short-term O_3_ exposure (60 μL/L for 48 h) on fresh grapes did not determine an immediate resveratrol accumulation, but it induced an elicitor effect on total stilbenes (+36%) in dehydrated grapes (20% of weight loss), with a considerable overproduction of *trans*-resveratrol and *trans*-piceatannol.

Furthermore, O_3_ treatments during grapes post-harvest seem to stimulate the berry skin cell wall degradation, affecting the extractability of oligomeric flavanols and proanthocyanidins [26]. As observed by Botondi et al. [11], O_3_ shock treatment on Pignola grapes did not affect the pectin methylesterase and polygalacturonase activities, which, in turn, affect cell wall composition and porosity, and are responsible for different anthocyanin and flavonol extractability [32]. Moreover, polyphenols are retained by the cell wall according to their structure. In this regard, the analysis of the texture has been proven to be an effective tool to correlate phenolic compounds extractability to skin mechanical properties. In particular, a significant correlation has been found between skin hardness and the extraction of anthocyanins and flavanols with low and high molecular mass [78]. Recently, Laureano et al. [79] observed a hardening of table (Italia and Muscat Hamburg) and wine (Merlot and Barbera) grapes’ berry skin as a consequence of post-harvest gaseous O_3_ exposure (30 μL/L) for 24 h, evidencing a role of O_3_ treatment on the berry skin mechanical features. The use of O_3_ for the treatment of wine grapes in post-harvest is currently being explored to improve polyphenol extractability, which is mainly affected by the cultivar and, to a lesser extent, by the time of the O_3_ exposure [26,30,77].

Lastly, the effect of O_3_ exposition (30 mg/L continuously supplied) on the aromatic composition of Moscato Bianco grapes during the dehydration process was investigated by Segade et al. [31]. O_3_ significantly increased not only the amount of total VOCs, but also the amount of terpenoids, which are the major aromatic markers of the Moscato scent. Accordingly, the biosynthetic pathways involved in terpenoids and C6 biosynthesis (i.e., MEP and LOX-HPL) were up-regulated following the O_3_ exposition. On the contrary, higher doses (60 mg/L) supplied for a shorter time significantly reduce total VOCs, due to terpenoids oxidation [14]. These findings highlight again that the effect of O_3_ on VOCs profile strongly depend on the concentration and exposure time.

## 5. Ozone Strategy to Increase Bioactive Compounds in Wine

With respect to wine production, the use of O_3_ has become popular, and its employment is increasing in a significant number of wineries. As already discussed, O_3_ has been widely used not only as sanitizing agent and for increasing the shelf-life of harvested grapes, but also as an exogenous elicitor to enrich grapes of secondary metabolites, significant factors for grape quality and human health. Hence, at berry level, the oxidative stress modifies the accumulation of different compounds to defend the cells from possible oxidative damages [73]. Most of the studies available in the literature focused on the elicitor effect on grape metabolism, confirming the aptitude of O_3_ in increasing the bioactive compound presence. However, when dealing with wine grapes, it is decisive to understand if the changes induced in grapes are transferred in the resulting wine, considering that the accumulation of those metabolites can increase wine health-promoting attributes thanks to their antioxidant activity [42].

As is obvious, the accumulation of secondary metabolites in grapes have a great influence on wine quality. For instance, modifications of the polyphenol profile in grapes will result in changes in color, astringency, bitterness, and body of the resulting wines. Furthermore, the accumulation of antioxidant volatiles, such as terpenoids and C6 compounds, induced by the oxidative stress [29,31,61] can strongly affect the aromatic profile of the wine. Nevertheless, very few studies on the accumulation of bioactive compounds in wine are currently available. 

### 5.1. Effect of Pre-Harvest O_3_ Application on Wine Features

Recently, O_3_ has been proposed for pest management in viticulture as a possible alternative to traditional pesticides, considering its environmental and human health friendliness. Studies related to the in-field O_3_ application on grapes, and associated to the wine quality and composition, are very limited. It is well established that viticulture practices (such as clusters thinning, defoliation, elicitor application, etc.) can strongly influence grape development and metabolism [58], and, consequently, wine composition. The main problem when dealing with pre-harvest applications is that O_3_ has always been considered as an environmental pollutant, associated with yield reduction, and the development of physiological disorders in plants (such as chlorophyll degradation, and a decrease of carbon and nitrogen assimilation) and fruits [80]. However, it is also clear that the possibility to cause damages is strongly affected by the cultivar, phenological stage, and environmental conditions. As such, there is a small risk of toxicity if O_3_ is applied under controlled conditions. Additionally, the controlled oxidative stress induces antioxidant responses which could enrich fruit and wine of secondary metabolites, exactly as they occur in post-harvest applications [29,72,81]. 

Recently, ozonated water was applied to control grapevine diseases, and the effect on grapes and wine quality (*Vitis vinifera* L. cv. Bobal) was studied by Campayo et al. [33,73,81]. These authors tested two different applications (i.e., a single treatment with ozonated water during the ripening period, and three treatments with ozonated water performed between fruit set-up to harvest), evaluating their effect on grapes, and during winemaking. The treatments induced a different response even within the same family of compounds. The first ozonated water treatment increased total polyphenol by about 130%. Concerning the effect of ozonated water on different classes of polyphenols, it is possible to observe how the single treatment led to a wine with higher (more than double of the control wine) phenolic acids, flavanols, flavonols, and anthocyanins. On the other hand, the trial performed using three ozonated water treatments increased stilbenes and flavanols, while reduced the amount of anthocyanins. Specifically, phenolic acids such as gallic, vanillic, syringic, *trans*-caftaric, and *trans*-*p*-coutaric acids were in higher amount after vines ozonation, regardless of the dose and type of application. Furthermore, among the different stilbenes identified by the authors, *trans*-resveratrol and glucoside piceid-*trans*-resveratrol increased in wine made starting from ozonated vines [33]. These compounds belong to the class of phytoalexins, and they are produced as defensive molecules in plants as a response to different stresses, as well as the oxidative stress [82]. Concerning flavanols, higher concentrations of catechin were found in wines made starting from ozonated vines. Flavanols are known as the most powerful ROS scavengers in grapes and wines, presenting an extremely high antioxidant activity [83], and, therefore, it is not surprising their increase in wines derived from ozonated vines. 

The amount of non-acylated anthocyanins (i.e., delphinidin, petunidin, peonidin, and malvidin 3-*O*-glucosides) and the peonidin and malvidin 3-*O*-glucosides acetylated increased in the wine made starting from ozonated vines. However, when the ozonated water treatments were repeated three times, the amounts of all the non-acylated anthocyanins decreased. The authors reported that these antioxidant compounds produced in grapes against oxidative stress would be oxidized and depleted under long exposure to ozone.

Regarding wine aroma, when plants are subjected to abiotic stresses, they increase the biosynthesis and emission of VOCs. Specifically, it is well known that plants produce isoprene, terpenes, and C6 in response to the oxidative stress after O_3_ exposition [37,38]. This was again confirmed by Campayo et al. [73], who observed an increase of the total free terpenoids in wine made starting from ozonated vine, mainly due to the accumulation of farnesol and nerolidol. When treatments were repeated during the growing season, citronellol and nerolidol increased. Based on recent studies, terpenoids have been demonstrated to induce health-beneficial effects, mainly for their anti-inflammatory and antimicrobial properties [84]. 

Based on the only one study available, it seems clear that the application of O_3_ on vines, by modifying grapes metabolism, increases the content of important health-related bioactive and antioxidant compounds in the resulting wine (i.e., polyphenols and terpenoids). 

### 5.2. Effect of Post-Harvest O_3_ Application on Wine Features 

Though the pre-harvest use of O_3_ is still very debated, its use for post-harvest management of wine grapes is widely used. Hence, in the context of new technologies for grapes preservation and wine making, O_3_ is currently a common tool not only to control spoilage microorganisms, but also to increase the nutritional value of grapes and wine. As already discussed in Section 4, there are many papers discussing the effect of post-harvest O_3_ treatment on grape composition and metabolic responses, whereas the effect of ozonation on wine quality is less studied. Many winemakers considered the use of O_3_ as a potential risk of oxidation of important compounds for the sensory and quality attributes of wine (i.e., polyphenols and volatiles). However, it is currently known that, if used under controlled conditions, it can represent a good option to increase the amount of these compounds. Hence, growing attention has been paid to O_3_, related to its stress action, which enhances the biosynthesis of bioactive compounds (such as phenolic substances) in table and wine grapes [20,28,85].

Mencarelli and Catelli [10] showed, for the first time, an enrichment of polyphenols and anthocyanins extraction in red wines treated with O_3_ according to the Purovino^®^ method (12 h, at 4 °C and 70% RH with max 20 g/h with 6% *w*/*w* of O_3_ and a flow rate at maximum 150 normal liter/h). The authors explain that the increase of these compounds (namely gallic acids, catechins, and epicatechins) is obtained thanks to two different, but linked, mechanisms [10]. On one hand, grapes exposition at a right dose of O_3_ for the right amount of time promotes biosynthesis of metabolites [10,14,86], and on the other hand, it modifies skin permeability, determining a consequent greater extraction of polyphenols. The treatment also promotes biosynthesis and extraction of antioxidant-related volatiles. Furthermore, the wines produced through the mentioned method were characterized by a polyphenols fraction which remained unaltered during malolactic fermentation and also in bottled wines (up to two years), indicating a greater stability of wines produced employing O_3_-treated grapes. Bellincontro et al. [15] observed an increase of anthocyanins and skin tannins in wine derived from O_3_-treated harvested grapes. The increase in anthocyanins is probably due to the berries’ reaction to a moderate stress, which induces polyphenols biosynthesis, which are then transferred in the resulting wine. 

Furthermore, after ozonation, the extractability index increased in O_3_-treated grapes, and, therefore, O_3_ treatment not only induces the biosynthesis of phenolic substances, but also affects the cell wall structure and cell membrane composition [87], facilitating the extraction of these compounds during winemaking. Ref. [11] thus represents a great advantage for the vinification process in term of time, cost, and health-related properties as well. 

The increase of polyphenols in wine grapes after post-harvest O_3_ exposition was also pointed out by Carbone and Mencarelli [28], Paissoni et al. [26], and Segade et al. [77], who reported an increase of catechin [28], and higher flavanol and anthocyanins content, thanks to a greater extraction [26,77]. The same increase of polyphenols, specifically of flavonoids, has been found even when O_3_ has been used at the beginning of controlled dehydration of Pignola and Romanesco grapes for “passito” wine production [11,29]. Lastly, post-harvest O_3_ treatments used to reduce the *smoke taint* in wine, which means wine made from grapes exposed to bushfires with undesirable sensory characters (smoky, burnt, and ashy), lead to higher polyphenol content as well [32,88]. Unfortunately, all these studies do not report any information about the resulting wines.

## 6. Conclusions

Overall, the O_3_ treatments operated on table and wine grapes reveal many important advantages, especially related to the phenolic and aromatic fractions. However, the effect of O_3_ treatment strongly depends on the treated cultivar, O_3_ form (gas, water), and method used for the treatment (i.e., dose, duration, intermittence or constant exposition). Both gaseous and water ozone treatment exert an elicitor effect on grape bioactive compounds (i.e., polyphenols, anthocyanins, flavanols, etc.), although most of the studies refer to gaseous treatments. Moreover, it is often reported that high doses and long exposition could result in an excessive oxidative stress which potentially decreases grape quality parameters, as bioactive compounds may be oxidized. On the other hand, when O_3_ is applied under studied and controlled conditions, an increase of bioactive compounds, such as polyphenols and antioxidant volatiles, is often reported. The literature suggests that in most of the cases, the best results are obtained with a low concentration and short treatment. Moreover, continuous ozone treatment during post-harvest dehydration increases the total VOCs. However, it is also suggested that the internal composition of the berries (cultivar-dependent) strongly influences the final result of O_3_ exposition. For example, in cultivars with higher flavanol and anthocyanins content, the result of O_3_ treatment is a greater extraction of polyphenols. Furthermore, in cultivars characterized by the prevalence of anthocyanins di-substituted, the result is a lower anthocyanin extractability, whereas in cultivars with tri-substituted anthocyanins, the extractability after O_3_ exposition is higher. 

In the light of all the above considerations, the factors affecting the bioactive compounds content in grapes, and, as a consequence, in wine, are many, and a unique strategy is, therefore, difficult to identify. Nevertheless, all considered, the potential role of O_3_ to stimulate the biosynthesis of bioactive compounds is clear, and, considering that O_3_ treatment is very practical, it can be easily incorporated into the wine production chain not only as sanitizing agent, but also to promote the health-related compounds of wines, inducing an improvement of their general quality. Moreover, post-harvest grape exposure could significantly reduce the use of sulphur dioxide in winemaking due to its bactericidal and fungicidal properties. However, in a large-scale application, especially in the case of winemaking goals, an adaptation of O_3_ treatment depending on the cultivar and on the target wine would also be highly desirable.

## Figures and Tables

**Table 1:** Effect of ozone treatment on bioactive compounds accumulation in table and wine grapes, and in wine made starting from ozonated grapes.

Product	Cultivar	Ozone Form	Ozone Treatment, Dose and Duration	^1^ Effect	Reference
Table grapes	Napoleón	Gas	38 days of storage at 0 °C under 0.1 mg/L of O_3_, and stored in 2.5 L glass jar with 8 mg/L of O_3_ for 30 min every 2.5 h	+ total stilbenes	Artés-Hernández et al., 2003 [19]
	Autumn Seedless	Gas	Continuous flow with 0.1 µL/L, and discontinuous with 8 µL/L for 30 min every 2.5 h	+ total flavanols + total hydroxycinnamic acid derivatives + total phenolics	Artés-Hernández et al., 2007 [20]
	Superior Seedless Regina Victoria Cardinal CL80	Gas	Continuous and discontinuous (12 h/days) O_3_ flows (2 mg/L) during 72 days of storage	+ resveratrol (with discontinuous flow)	Cayuela et al., 2009 [21]
	Scarlotta	Gas	Pre-storage treatment with 5, 10, and 20 μL for 30 min at 0 °C	+ total polyphenols + antioxidant activity + total anthocyanin	Admane et al., 2018 [22]
	Seedless Black grapes	Water	Immersion in pre-storage with ozonated water (2, 4, 6, or 8 mg/ L) for 4 min at 5 °C	+ total polyphenols + antioxidant activity	Silveira et al., 2018 [23]
	Perlette Thompson Zeiny Alphonse Lavallee Barlinka	Gas	Pre-storage treatment with 16 mg/L for 5 to 10 min	+ phytoalexins (resveratrol, pterostilbene)	Sarig et al., 1996 [24]
	Superior	Gas	Pre-storage treatment with 1.67 and 3.88 g/h 1, 3, and 5 h	+ total stilbenes	González-Barrio et al., 2006 [25]
Wine grapes	Barbera Nebbiolo	Gas	Post-harvest treatment pre-vinification for 24 and 72 h with 30 μL/L	+ total proanthocyanidins extraction + flavanols extraction + total anthocyanin extraction	Paissoni et al., 2017 [26]
	Petit Verdot	Gas	Post-harvest treatment pre-vinification for 12 h with 20 g/h	+ total anthocyanin	Bellincontro et al., 2017 [15]
	Maturano	Gas	Pre-harvest treatment	+ chlorogenic acid	Valletta et al., 2016 [27]
	Grechetto	Gas	Post-harvest treatment pre-vinification for 12 h with 1.5 g/h	+ catechins	Carbone and Mencarelli 2015 [28]
	Pignola	Gas	Post-harvest treatment pre-vinification for 18 h with 1.5 g/h	+ polyphenols ± carotenoids ± anthocyanin + anthocyanin extractability + flavonol extractability	Botondi et al., 2015 [11]
	Romanesco	Gas	Post-harvest treatment pre-dehydration process with 20 g/h for 12 h	+ polyphenols	Modesti et al., 2018 [29]
	Nebbiolo	Gas	Constant flow during dehydration process with 30 µL/L	± total anthocyanin − anthocyanins extraction	Rìo Segade et al., 2020 [30]
	Barbera	Gas	Constant flow during dehydration process with 30 µL/L	− total anthocyanin + anthocyanins extraction	Rìo Segade et al., 2020 [30]
	Moscato bianco	Gas	Pre-dehydration treatment with 60 mL/L for 24 or 48 h	+ glycosylated VOCs + free linalool + cis-furan linalool oxide + terpineol	Rìo Segade et al., 2018 [14]
	Moscato bianco	Gas	Constant flow during dehydration process with 30 µL/L	+ glycosylated VOCs + linalool + geraniol + nerol	Rìo Segade et al., 2017 [31]
	Merlot	Gas	Post-harvest treatment with 1 and 3 mg/L for 12 and 24 h	+ total polyphenols	Modesti et al., 2021 [32]
Wine	Bobal	Water	Pre-harvest singles treatment	+ total polyphenols + phenolic acids + flavanols + flavonols + anthocyanins + free terpenoids	Campayo et al., 2020 [33]
	Bobal	Water	Pre-harvest treatments (three treatments performed between fruit set and harvest)	+ phenolic acids + flavanols + stilbenes + farnesol + nerodiol	Campayo et al., 2020 [33]
	Petit Verdot	Gas	Post-harvest treatment pre-vinification for 12 h with 20 g/h	+ anthocyanins + skin tannins	Bellincontro et al., 2017 [15]
Patent	PCT/IB2012/000036 “Process for the Treatment and the Winemaking of Grapes”	Gas	Post-harvest treatment with 20 g/h for 12 h	+ gallic acids + catechins + epicatechins	Mencarelli and Catelli, 2012 [10]

^1^ Notes: + = increase; − = decrease; ± = both increase or decrease.
